# Effect of marker segregation distortion on high density linkage map construction and QTL mapping in Soybean (*Glycine max* L.)

**DOI:** 10.1038/s41437-019-0238-7

**Published:** 2019-05-31

**Authors:** Jian-Fang Zuo, Yuan Niu, Peng Cheng, Jian-Ying Feng, Shi-Feng Han, Ying-Hao Zhang, Guoping Shu, Yibo Wang, Yuan-Ming Zhang

**Affiliations:** 1 Crop Information Center, College of Plant Science and Technology, Huazhong Agricultural University, Wuhan, 430070 China; 2 College of Life Sciences and Food Engineering, Huaiyin Institute of Technology, Huaian, 223003 China; 3 State Key Laboratory of Crop Genetics and Germplasm Enhancement, Nanjing Agricultural University, Nanjing, 210095 China; 4 Center of Molecular Breeding and Biotechnology, Beijing Lantron Seed Corp., Beijing, 100081 China

**Keywords:** Genetic linkage study, Plant hybridization

## Abstract

Marker segregation distortion is a natural phenomenon. Severely distorted markers are usually excluded in the construction of linkage maps. We investigated the effect of marker segregation distortion on linkage map construction and quantitative trait locus (QTL) mapping. A total of 519 recombinant inbred lines of soybean from orthogonal and reciprocal crosses between LSZZH and NN493-1 were genotyped by specific length amplified fragment markers and seed linoleic acid content was measured in three environments. As a result, twenty linkage groups were constructed with 11,846 markers, including 1513 (12.77%) significantly distorted markers, on 20 chromosomes, and the map length was 2475.86 cM with an average marker-interval of 0.21 cM. The inclusion of distorted markers in the analysis was shown to not only improve the grouping of the markers from the same chromosomes, and the consistency of linkage maps with genome, but also increase genome coverage by markers. Combining genotypic data from both orthogonal and reciprocal crosses decreased the proportion of distorted markers and then improved the quality of linkage maps. Validation of the linkage maps was confirmed by the high collinearity between positions of markers in the soybean reference genome and in linkage maps and by the high consistency of 24 QTL regions in this study compared with the previously reported QTLs and lipid metabolism related genes. Additionally, linkage maps that include distorted markers could add more information to the outputs from QTL mapping. These results provide important information for linkage mapping, gene cloning and marker-assisted selection in soybean.

## Introduction

Soybean (*Glycine max* L. Merr., 2*n* = 40) is an important source of protein and oil for food and feed. Linkage map plays an important foundational role in soybean genetics and molecular biology, such as quantitative trait locus (QTL) mapping and candidate gene mining, map-based cloning, whole-genome *de novo* assembly, marker-assisted selection, and genome selection (Xia et al. 2007). As we know, marker segregation distortion is a common phenomenon in linkage map construction and QTL mapping. In most research and applications, significantly distorted markers are either simply discarded or included without notice and their impact on high density linkage map construction and QTL mapping is largely unknown.

To date, many studies on linkage map construction in soybean have been reported. Initially, the linkage maps were constructed using morphology and isoenzyme markers, e.g., the first soybean genetic map was constructed by 57 classical markers (Palmer and Kilen 1987). Clearly, the number of markers was too small. With the development of more advanced technologies, molecular markers have been used to construct linkage maps. These markers have the potential to “saturate” the genome and this increases the probability of QTL detection (Keim et al. 1990). Although these markers are useful in the construction of high density linkage maps, large gaps occur frequently (Wu et al. 2001). Thus, integrated linkage maps have been often reported in soybean (Cregan et al. 1999; Yamanaka et al. 2001; Song et al. 2004; Xia et al. 2007), e.g., all the 165 RFLP, 25 RAPD and 650 AFLP markers in Keim et al. (1997), and all the 1015 SSR, 709 RFLPs, 73 RAPDs, 6 AFLPs and 46 other markers in Song et al. (2004). With the development of genomic sequencing technology in recent years, several thousand single nucleotide polymorphism (SNP) markers have become available to construct linkage maps (Choi et al. 2007; Hyten et al. 2010; Song et al. 2016). Recently, Collard and Mackill (2008) established, and Sun et al. (2013) described in detail, specific length amplified fragment sequencing (SLAF-seq) technology, as a high-resolution strategy, for high-throughput SNP genotyping. These SLAF markers have been frequently used to construct linkage maps. In soybean, Zhang et al. (2013), Qi et al. (2014) and Li et al. (2014) used 1233, 5308 and 5785 SLAF markers, respectively, to construct their high-density linkage maps. In the above studies, however, severely distorted markers were not included in their linkage maps.

Marker segregation distortion, defined as the significant deviation of the observed segregation ratio from the Mendelian ratio expected from the mating design of the studied population, is a common biological phenomenon (Lyttle 1991). To date, there have been many studies on this research topic (Wang et al. 2005; Zhu et al. 2007; McMullen et al. 2009; Gardner et al. 2016). Hackett and Broadfoot (2003) showed that segregation distortion has very little effect on marker order or length when the distances between adjacent markers are approximately 10 cM. However, the average genetic distance in the currently reported linkage maps is frequently less than 1 cM. Thus, this issue needs to be further studied.

In this study, five hundred and nineteen recombinant inbred lines (RILs) from orthogonal and reciprocal crosses of soybean cultivars LSZZH (P_1_) with NN493-1 (P_2_) were genotyped by SLAF markers and the seed linoleic acid content was measured in three environments. All the SNP markers, including very significantly distorted markers, were used to construct a high-density linkage map. In addition, the effect of these distorted markers on linkage map construction and QTL mapping was investigated. To validate the effectiveness of constructed linkage maps in this study, we evaluated the quality of the linkage maps, and detected QTLs for seed linoleic acid content in soybean.

## Materials and methods

### Plant materials and DNA extraction

Five hundred and nineteen recombinant inbred lines (RILs) derived from the orthogonal (242, OC) and reciprocal (277, RC) crosses between two parents LSZZH (P_1_) and NN493-1 (P_2_), together with their parents, were planted in three-row plots in a completely randomized design at the Jiangpu experimental station of Nanjing Agricultural University in 2015 (NJ2015), and at the Wuhan and Ezhou experimental stations of Huazhong Agricultural University, respectively, in 2014 (WH2014) and 2015 (EZ2015). The plots were 1.5 m wide and 2.0 m long, and approximately 15 plants were planted in each row. Five plants in the middle row for each line were randomly harvested, and 10 g of seeds were prepared for the measurement of linoleic acid content.

The young healthy leaves from 519 RILs as well as from their two parents were collected and frozen in liquid nitrogen. Total genomic DNA was extracted from each leaf sample using the cetyltrimethyl ammonium bromide (CTAB) method (Doyle 1990). All the information obtained from 519 RILs and their parents was used for genotyping and mapping analyses.

### Genotyping the RIL population with the SLAF-seq method

SLAF-seq technology, developed by the Beijing Biomarker Technology Corporation (Collard and Mackill 2008), was used to genotype 519 RILs and the two parents in this study. The sequencing and preliminary bioinformatics analyses were as described by Sun et al. (2013) with minor modifications. The soybean reference genome (*Glycine max* Wm82.a1.v1) was used to align the SLAF markers for SNP discovery. The SLAF library construction was performed accordingly using the predesigned scheme. Genomic DNA from each sample was digested by two restriction enzymes, *RsaI* (NEB, Ipswich, MA, USA) and *HaeIII* enzyme. Then, restriction-ligation samples were diluted and mixed with dNTP, Taq DNA polymerase (NEB) and primer for PCR reactions. The PCR productions were purified using E.Z.N.A.® Cycle Pure Kit (Omega) and pooled. The pooled samples were incubated with MseI, T4-DNAligase, ATP and Solexa adapter at 37°C, then purified with a Quick Spin column (Qiagen, Hilden, Germany), and electrophoresed on a 2% agarose gel. After gel purification, DNA fragments 314–414 bp in length with indices and adaptors, defined as SLAF tags, were excised and diluted for pair-end sequencing on an Illumina high-throughput sequencing platform (Illumina, Inc.; San Diego, CA, US).

The SLAF-seq data grouping and genotyping were described in detail by Sun et al. (2013). Based on sequence similarity, all the SLAF pair-end reads with clear index information were clustered. To reduce computational intensity, identical reads were merged together, and sequence similarity was detected using one-to-one alignment by BLAT (tileSize = 10, stepSize = 5) (Kent 2002). Sequences with over 90% identity were grouped to one SLAF locus. Through the minor allele frequency (MAF) evaluation, alleles were defined in each SLAF marker. Because soybean is a diploid species, one locus can contain no more than four SLAF tags; groups containing over four tags were considered to be repetitive SLAFs and were filtered out. Alleles of each SLAF locus were then defined according to the average sequence depths of SLAF markers, which were greater than 10-fold in parents and greater than 3-fold in RILs. High-quality SLAF markers for the genetic mapping were filtered by the following criteria: (i) average sequence depths should be > 10-fold in the parents, (ii) markers with more than 25% missing data were excluded, and (iii) the loci containing more than four SLAF tags were excluded.

### Linkage map construction

The program MSTmap (Wu et al. 2008) was used to group and order the SLAF markers. Considering the existence of marker segregation distortion, the software DistortedMap of Xie et al. (2014) was used to detect marker segregation distortion. The quality of the constructed linkage maps was evaluated by the collinearity between the linkage maps in this study and the soybean reference genome, the heat map and the uniform distribution of recombination fractions on the genome. The collinearity was measured by the Spearman correlation coefficient, which was calculated by the R function *cor.test*. The higher the Spearman correlation coefficient, the better the collinearity. The R program *pheatmap* (https://cran.r-project.org/web/packages/pheatmap/index.html) was used to construct the heat maps.

### Linoleic acid content measurement

In WH2014, EZ2015 and NJ2015, approximately 10 g of seeds collected from five plants per RIL and the two parents were ground using a pulverizer, and the seed powder was filtered. Then, 30 mg soybean powder was used to extract fatty acid. Five fatty acids for each line were measured by gas chromatography with a flame ionization detector and a Permabond FFAP stainless steel column (50 m × 0.2 mm × 0.33 µm, ThermoFisher Scientific, Waltham, MA) at the Wuhan Research Branch of the National Rapeseed Genetic Improvement Center in 2014 and 2015, respectively, and the details were described by Zhou et al. (2016).

### Genome-wide composite interval mapping of QTLs for linoleic acid content

The linoleic acid contents for each RIL in the three environments, WH2014, EZ2015 and NJ2015, were indicated by datasets I, II and III, respectively. The BLUP values (dataset IV) were predicted by the R program *lme4* (https://CRAN.R-project.org/package=lme4).

All the above four datasets for seed linoleic acid content, along with marker genotypic information and linkage maps, were used to detect QTLs using genome-wide composite interval mapping (Wang et al. 2016), implemented by the QTL.gCIMapping.GUI program (https://cran.r-project.org/web/packages/QTL.gCIMapping.GUI/index.html). The covariate on the orthogonal and reciprocal crosses was included in the genetic model while all the 519 RILs were jointly analyzed. The walk speed for genome-wide scanning was set at 1 cM. The LOD score thresholds for significant QTLs at the 0.05 probability level were calculated based on 1000 permutations using the Windows QTL Cartographer v2.5 software (Wang et al. 2012). The QTLs with the LOD scores between the threshold and 2.5 were viewed as suggestive QTLs (Lander and Kruglyak 1995).

The QTLs, detected repeatedly across the above four datasets, were viewed as stable. If the QTLs identified across various datasets were within 5 cM, these QTLs were viewed as being the same (Song et al. 2004). If the QTL region in the linkage maps including distorted markers (case I) overlapped, in the physical positions of the genome, with ones excluding distorted markers (case II), these QTLs were also viewed as being the same. The nomenclature for detected QTL was denoted as *q* + trait name + chromosome + the number of QTL on the chromosome, such as “*qLA1-1*”, “*qLA*” indicated one QTL for linoleic acid content in soybean, and “*1-1*” indicates the first QTL on chromosome 1 (McCouch et al. 1997).

Previously reported QTLs from https://www.soybase.org/ and 1123 lipid metabolism related genes in Zhang et al. (2016) were used to identify the true QTLs for seed linoleic acid content in soybean. These true QTLs were used to validate the correctness of the linkage maps in this study.

## Results

### Genotyping of RIL population using the SLAF-seq method

**Fig. 1:**
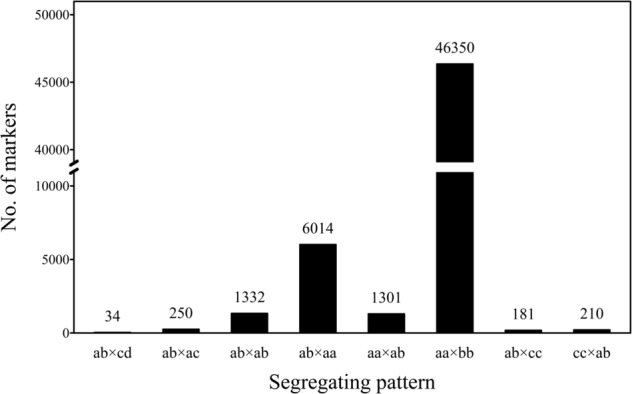
Number of markers with various segregation patterns in soybean. ab × cd, ab × ac, ab × ab, ab × aa, aa × ab, aa × bb, ab × cc and cc × ab are segregation patterns of markers, where the English alphabets from a to d are the alleles for each marker

Using the SLAF-seq method, a total of 384.64 M of raw data was generated; this comprised 22,595,092 reads for the female parent, 31,069,592 reads for the male parent, and 635,267 reads on average for each RIL. After clustering of the reads with their reference, a total of 418,100 SLAF labels were obtained from all the RILs and two parents. More specifically, 392,904 SLAFs were generated from 21,133,891 reads for the male (average sequence depth 53.79-fold), 391,472 SLAFs from 14,542,086 reads for the female (37.15-fold), and 165,417 SLAFs from 427,897 reads on average for each RIL (2.57-fold). Among the 418,100 SLAF labels, 58,351 (13.96%) were polymorphic. All of the SLAF labels were mapped on the soybean reference genome (*Glycine max* Wm82.a1.v1) using SOAP software (Li et al. 2008), 411,262 were distributed on 20 chromosomes (Fig S1), and 6838 were on a scaffold. After filtering out the SLAF markers lacking parent information, 55,672 were retained and classified into eight types (Fig. 1). In the RIL population, only the 46,350 SLAF markers with the aa × bb segregation pattern were used to construct the linkage map (Fig. 1). In order to ensure the quality of our linkage map, the SLAF labels with an average sequence depth <10-fold in the parents, >25% missing data, and >4 SLAF tags, were filtered out. Among the 11,979 SLAF labels finally used in this study, 11,846 were mapped on 20 chromosomes, and 133 were on a scaffold.

### Construction of a high-density genetic linkage map in soybean

#### Construction of linkage maps in the RILs from the orthogonal and reciprocal crosses

With OC RILs, all the 11,846 markers covered 2631.89 cM on 20 soybean chromosomes with an average marker-interval of 0.223 cM; total map length for each chromosome ranged from 68.13 cM (chr14) to 206.21 cM (chr2), and chromosomes 14 and 5 had the minimum (0.14 cM) and maximum (0.71 cM) average marker-intervals, respectively; the maximum (18.68 cM) and minimum (2.49 cM) marker-gaps were found on chromosomes 19 and 14, respectively (Table 1).

**Table 1:** Description of basic characteristics for 20 linkage groups in orthogonal cross, reciprocal cross and all the BRILs

Chr.	No. of markers	All the RILs				Orthogonal cross RILs				Reciprocal cross RILs			
		Total genetic distance (cM)	Average genetic distance (cM)	Maximum gap (cM)	Collinearity^a^	Total genetic distance (cM)	Average genetic distance (cM)	Maximum gap (cM)	Collinearity^a^	Total genetic distance (cM)	Average genetic distance (cM)	Maximum gap (cM)	Collinearity^a^
1	507	150.48	0.30	7.14	0.9962	147.36	0.29	5.72	0.9831	158.33	0.31	8.41	0.9857
2	1116	176.26	0.16	4.84	0.8705	206.21	0.18	5.39	0.8737	177.20	0.16	4.98	0.8848
3	917	137.01	0.15	6.9	0.9372	146.49	0.16	5.72	0.9271	149.49	0.16	7.99	0.8773
4	277	80.78	0.29	3.34	0.9406	79.27	0.29	4.14	0.9387	86.63	0.31	4.18	0.9392
5	152	107.95	0.71	4.24	0.9339	107.96	0.71	4.75	0.9356	107.92	0.71	4.67	0.9338
6	466	158.82	0.34	4.05	0.9998	164.10	0.35	4.10	0.9995	169.70	0.36	4.14	0.9987
7	637	153.64	0.24	4.47	0.9645	157.59	0.25	5.72	0.9545	177.66	0.28	5.7	0.9534
8	229	100.04	0.44	2.71	0.9991	101.29	0.44	3.20	0.9991	104.72	0.46	3.12	0.9990
9	917	158.93	0.17	5.16	0.9850	159.14	0.17	5.02	0.9551	196.18	0.21	5.29	0.8997
10	669	149.87	0.22	4.21	0.8214	155.36	0.23	4.34	0.6557	154.13	0.23	4.1	0.7813
11	354	80.02	0.23	3.41	0.8414	79.70	0.23	3.45	0.8208	85.53	0.24	3.38	0.8157
12	163	82.72	0.51	6.22	0.9992	74.00	0.45	5.86	0.9988	85.45	0.52	5.24	0.9992
13	646	159.15	0.25	13.32	0.9999	185.81	0.29	15.88	0.9897	163.71	0.25	11.22	0.9889
14	499	67.07	0.13	2.78	0.9883	68.13	0.14	2.49	0.9858	79.26	0.16	3.72	0.9879
15	1327	147.50	0.11	5.96	0.9431	183.80	0.14	6.51	0.8918	159.18	0.12	5.51	0.8488
16	670	119.53	0.18	3.97	0.8950	138.46	0.21	3.48	0.8837	118.09	0.18	4.41	0.8669
17	1168	150.68	0.13	4.78	0.9550	183.79	0.16	4.47	0.8889	154.71	0.13	5.39	0.8095
18	610	136.60	0.22	7.75	0.9926	139.83	0.23	8.23	0.9901	154.69	0.25	6.93	0.9933
19	236	77.81	0.33	17.03	0.9961	77.81	0.33	18.68	0.9941	77.27	0.33	15.75	0.9950
20	286	81.01	0.28	4.24	0.9968	75.81	0.27	4.61	0.9964	83.40	0.29	4.18	0.9953
Total	11846	2475.86	0.21	17.03		2631.89	0.22	18.68		2643.24	0.22	15.75	

^a^The collinearity is measured by correlation coefficient

With RC RILs, all the 11,846 markers covered 2643.24 cM across 20 soybean chromosomes with an average marker-interval of 0.224 cM; total map length for each chromosome ranged from 77.27 cM (chr19) to 196.18 cM (chr9), and chromosomes 15 and 5 showed the minimum (0.12 cM) and maximum (0.71 cM) average marker-intervals, respectively; the maximum (15.75 cM) and minimum (3.12 cM) marker-gaps were found on chromosomes 19 and 8, respectively (Table 1).

**Fig. 2:**
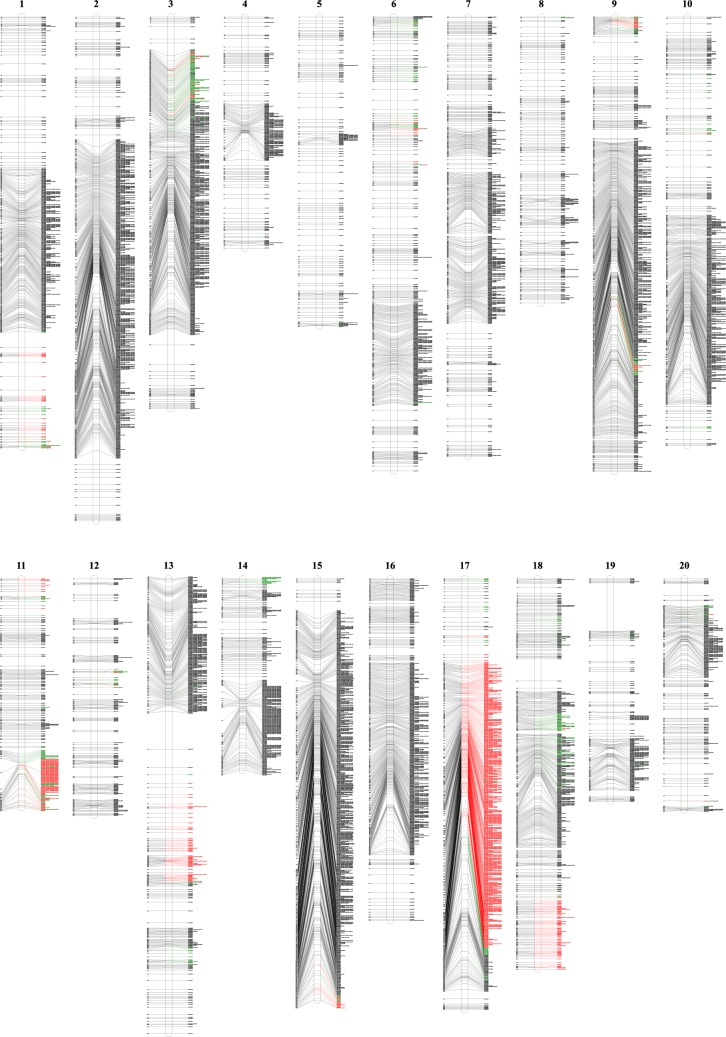
High-density genetic linkage maps constructed by 11846 markers in all the RILs from the orthogonal and reciprocal crosses between LSZZH and NN493-1 in soybean. The significantly distorted markers at the 0.05 and 0.01 probability levels are marked by green and red colors, respectively

With all the RILs, all the 11,846 markers covered 2475.86 cM across 20 soybean chromosomes with an average marker-interval of 0.209 cM; the number of markers for each chromosome ranged from 152 (chr5) to 1327 (chr15); total map length for each chromosome ranged from 67.07 cM (chr14) to 176.26 cM (chr2), and chromosomes 15 and 5 showed the minimum (0.11 cM) and maximum (0.71 cM) average marker-intervals, respectively; the maximum (17.03 cM) and minimum (2.71 cM) marker-gaps were found on chromosomes 19 and 8, respectively (Table 1; Fig. 2).

#### Effect of marker segregation distortion on linkage map construction

The software DistortedMap was used to detect marker segregation distortion. As a result, 2306, 2431 and 1999 markers showed the distortion from Mendelian segregation in the OC, RC and all the RILs, respectively (Table 2). Among these distorted markers, 750, 609 and 486 in the OC, RC and all the RILs, respectively, were significant (0.01 < *P*-value ≤ 0.05), and 1556, 1822 and 1513 were very significant (*P*-value ≤ 0.01). As shown in Table 2, more than 100 distorted markers with *P*-values ≤ 0.05 were observed on chromosomes 2 (131), 15 (128), 17 (1130), and 18 (394) in the OC RILs, on chromosomes 2 (914), 6 (116), 11 (265), 13 (461) and 19 (222) in the RC RILs, and on chromosomes 3 (107), 11 (255), 17 (1097) and 18 (175) in all the RILs. More than 100 distorted markers with *P*-values ≤ 0.01 were observed on chromosomes 17 (1107, 94.78%) and 18 (240) in the OC RILs, on chromosomes 2 (857, 76.79%), 6 (103), 11 (228) and 13 (405) in the RC RILs, and on chromosomes 11 (187) and 17 (1070, 91.61%) in all the RILs. Among these distorted markers, 63 and 18 were common between the orthogonal and reciprocal crosses at the 0.05 and 0.01 probability levels, respectively; 53 and 15 were common across the above three populations at the 0.05 and 0.01 probability levels, respectively.

**Table 2:** Information on segregation distorted markers in orthogonal cross, reciprocal cross and all the RILs

Chr.	No. of markers	All the RILs			Orthogonal cross RILs			Reciprocal cross RILs		
		0.01 < *P* < 0.05	*P* ≤ 0.01	Total	0.01 < *P* < 0.05	*P* ≤ 0.01	Total	0.01 < *P* < 0.05	*P* ≤ 0.01	Total
1	507	20	28	48	18	29	47	2	0	2
2	1116	0	0	0	122	9	131	57	857	914
3	917	89	18	107	6	1	7	90	2	92
4	277	2	0	2	0	0	0	7	0	7
5	152	3	0	3	28	9	37	9	0	9
6	466	44	13	57	1	0	1	13	103	116
7	637	1	0	1	24	3	27	9	10	19
8	229	2	0	2	45	27	72	13	1	14
9	917	33	22	55	70	20	90	0	0	0
10	669	22	1	23	60	6	66	14	10	24
11	354	68	187	255	0	0	0	37	228	265
12	163	13	3	16	19	14	33	24	9	33
13	646	20	76	96	27	9	36	56	405	461
14	499	24	0	24	22	9	31	2	0	2
15	1327	7	13	20	62	66	128	20	9	29
16	670	0	0	0	10	0	10	50	0	50
17	1168	27	1070	1097	23	1107	1130	15	0	15
18	610	95	80	175	154	240	394	14	80	94
19	236	4	0	4	38	1	39	130	92	222
20	286	12	2	14	21	6	27	47	16	63
Total	11846	486	1513	1999	750	1556	2306	609	1822	2431

The program MSTMap (Wu et al. 2008) was used to construct linkage maps using marker datasets that include or exclude the distorted markers in the OC, RC and all the RILs. With the marker datasets that include distorted markers, all the 11846 markers were grouped into 20 linkage groups and each linkage group across different populations has the same number of markers (Table 1). With the marker datasets that exclude all the very significantly distorted markers with *P*-values ≤ 0.01, 10,290 markers in OC RILs were grouped onto 22 linkage groups, because 2 and 4 markers on chromosomes 15 and 17 were clustered onto two additional linkage groups; 10,024 markers in RC RILs were grouped onto 23 linkage groups, because 34, 4 and 9 markers on chromosomes 2, 6 and 11 were clustered onto three additional linkage groups; 10,333 markers in all the RILs were grouped onto 23 linkage groups, because 93 markers on chromosome 13, and 2 and 24 markers on the chromosome 17 were clustered onto three additional linkage groups (Table S1, Fig S2). We compared the above two kinds of linkage maps (including or excluding the very significantly distorted markers with *P*-value ≤ 0.01). First, in most cases the marker orders of linkage maps excluding the very significantly distorted markers were consistent with those including distorted markers. However, we found three inconsistent regions on chromosome 2 in OC RILs and on chromosome 10 in RC RILs (Fig S2). On chromosome 2, there were two inconsistent regions. The first region included 217 markers, which were between Marker1198674 (56.48 and 54.98 cM on the linkage maps including and excluding the very significantly distorted markers, respectively) and Marker1293705 (79.59 and 78.51 cM). The second region included 585 markers, which were between Marker1187344 (83.57 and 82.49 cM) and Marker1290774 (129.61 and 132.51 cM). On chromosome 10, there was one inconsistent region. This region included 444 markers, which were between Marker1053787 (84.00 and 83.82 cM) and Marker1019033 (112.14 and 111.22 cM).

If all the very significantly distorted markers with *P*-value ≤ 0.01 are excluded, then the total number of markers changed from 11,846 to 10,333, 10,290 and 10,024 in all, OC and RC RILs, respectively. The total genetic distances changed from 2475.86 to 2336.74 cM in all the RILs, from 2631.89 to 2464.72 cM in the OC RILs, and from 2643.24 to 2495.10 cM in the RC RILs. The average marker-interval changed from 0.21 to 0.23 cM in all the RILs, from 0.22 to 0.24 cM in the OC RILs, and from 0.22 to 0.25 cM in the RC RILs (Table S1). The above changes are mainly derived from a few chromosomes, e.g., the numbers of markers changed from 1168 to 98 and 61 on chr17 in the all and OC RILs, respectively, and from 1116 to 259 on chr2 in the RC RILs. The total genetic distances reduced from 150.68 and 183.79 to 84.61 and 58.37 cM on chr17 in the all and OC RILs, respectively, and from 177.20 to 137.83 cM on chr2 in the RC RILs. The average marker-interval reduced from 0.13 and 0.16 to 0.86 and 0.96 cM on chr17 in the all and OC RILs, respectively, and from 0.16 to 0.53 cM on chr2 in the RC RILs. This means that more markers increase total genetic distance and marker density on the chromosome.

In addition, we found one interesting phenomenon on chr2. Although one hundred and thirty-one (9) and 914 (857) markers had (highly) significant segregation distortion from the Mendelian ratio in the OC and RC RILs, respectively, no marker segregation distortion was observed in all the RILs (Table 2).

### The evaluation of linkage map constructed from all the RILs

#### The collinearity of linkage maps with soybean reference genome

To evaluate the collinearity between linkage maps and soybean reference genome, the Spearman correlation coefficient for each chromosome was calculated. The results are listed in Table 1. Among all the twenty coefficients in the OC RILs, 14 were larger than 0.90, 6 were larger than 0.99, and their range was from 0.6557 (chr10) to 0.9995 (chr6). Among these coefficients in the RC RILs, 12 were larger than 0.90, 6 were larger than 0.99, and their range was from 0.7813 (chr10) to 0.9992 (chr12). Among these coefficients in all the RILs, 16 were larger than 0.90, 8 were larger than 0.99, and their range was from 0.8214 (chr10) to 0.9999 (chr13). In addition, the consecutive curves between soybean genome and linkage groups are found in Fig S3. The high collinearity suggests that the genetic linkage maps we constructed are congruent with the soybean genome for marker orders.

If all the very significantly distorted markers with *P*-values ≤ 0.01 are excluded, in most cases the Spearman correlation coefficients between linkage maps and soybean reference genome were slightly reduced, and special situations were found for chromosomes 2, 11 and 17 in the RC, all and OC RILs, respectively (Table S2). The three relatively high collinearities were derived from very small (259, 167 and 61) markers, because 857, 187 and 1107 markers were excluded in the above three cases.

#### Heat map

To assess the quality of the linkage map, heat maps were generated using pair-wise recombination fractions (*r*) for the 11,846 SLAF markers (Fig S4). The size for *r* was indicated by different colors ranging from yellow (lower) to purple (higher). As shown in Fig S4, the colors on and near diagonal lines for all the chromosomes are yellow, indicating their lower recombination or high linkage disequilibrium, and the squares of different size along the diagonal lines indicates the existence of LD blocks of different size.

#### The recombination pattern in soybean genome

Each chromosome was divided into 20 intervals based on their physical positions. In each interval, the recombinant fraction between adjacent markers was obtained, and the sum of all the recombinant fractions in this interval was viewed as the recombinant fraction of this interval. The results are shown in Fig.S5. In Fig. S5, the recombination fractions were high in the intervals near the two ends of each chromosome and low in the intervals of middle region of each chromosome. This result is consistent with those in previous studies.

### Multi-QTL mapping for linoleic acid content in soybean

#### Mapping QTL for seed linoleic acid content in soybean

The linoleic acid content of LSZZH was larger than NN493-1 in all the environments, and there exists large variation among all the RILs in all the environments, e.g., the linoleic acid contents ranged from 40.64 to 57.70 (%) in WH2014. Similar phenomena were also observed in the other three phenotypic datasets (Table S3). The frequency distributions for the four phenotypic datasets are shown in Fig S6.

**Fig. 3:**
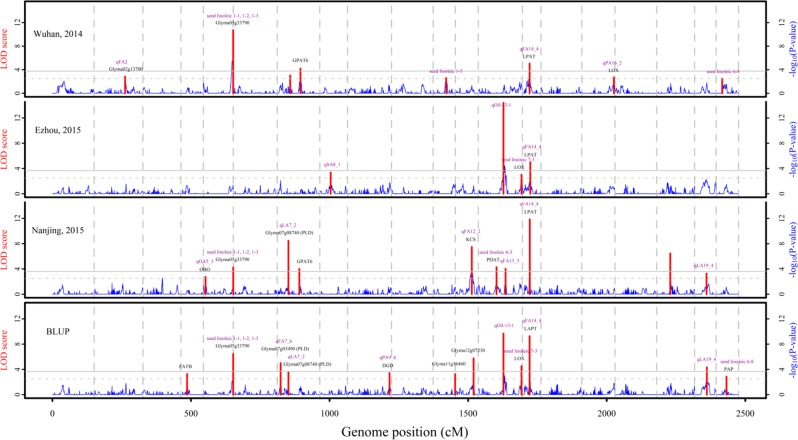
QTLs for soybean seed linoleic acid content in Wuhan, Ezhou, Nanjing and the BLUP values from linkage maps that include very significantly distorted markers using QTL.gCIMapping.GUI. The –log_10_(*P*-value) and LOD score of QTLs were marked respectively by the blue and red lines. Gray solid and dotted horizontal lines were the LOD thresholds respectively for significant and suggestive QTLs. Previously reported QTLs and lipid metabolism related genes are marked by dark magenta and black colors, respectively

If the linkage maps including very significantly distorted markers (case I) were used to conduct QTL mapping, the LOD score thresholds at the 0.05 probability level were 3.77, 3.66, 3.60 and 3.67, respectively, for the datasets I to IV. A total of 13 significant QTLs were detected. Among these 13 QTLs, 3, 2, 8 and 7 were found to be associated with linoleic acid content, respectively, for the datasets I to IV; 10 were previously reported; 9 were around the lipid metabolism related genes in Zhang et al. (2016) (Fig. 3, Table 3 and S4). In addition, a total of 11 suggestive QTLs were identified. Among these 11 suggestive QTLs, 5, 2, 8 and 7 were found to be associated with linoleic acid content, respectively, for the datasets I to IV; 8 were previously reported; 7 were around the lipid metabolism related genes in Zhang et al. (2016) (Fig. 3, Table 3 and S4). In summary, a total of 24 QTLs were identified, 18 were previously reported QTLs and 16 were around the lipid metabolism related genes (Fig. 3 and Table S4).

**Table 3:** The linoleic acid content QTLs detected by GCIM and confirmed by both previously reported QTLs in soybean and/or lipid metabolism related genes

QTL	Chr	QTLs detected							Previously reported QTLs		Lipid metabolism related genes around QTLs	
		Position (cM)	Marker intervals	LOD	Effect	*r*^2^ (%) ^a^	Environment ^b^	Type	QTL	Reference	Gene ^c^	Reference
*qLA2-1*	2	111.81	Marker1183683	2.85	−0.54	2.11	WH2014	Suggestive	*qFA2*	Li et al. 2017	*Glyma02g13700*	
*qLA4-1*	4	22.26	Marker2230222	3.28	0.31	1.99	BLUP	Suggestive			*Glyma04g37420* (*FATB*)	Salas and Ohlrogge 2002; Ozseyhan et al. 2018
*qLA5-1*	5	7.97	Marker2204980	2.74	0.41	1.75	NJ2015	Suggestive	*qOA5_3*	Li et al. 2017	*Glyma05g07880* (*OBO*)	Wang et al. 2008
***qLA5-2***	5	107.75; 107.95	Marker2104599; Marker2161702	6.47; 4.24; 10.72	0.43; 1.07; 0.52	3.84; 8.25; 2.77	BLUP; H2014; NJ2015	Significant	*seed linoleic 1-1, 1-2, 1-3*	Diers and Shoemaker (1992); Li et al. (2017); Cao et al. (2017)	*Glyma05g33790*	Chen et al. 2018
***qLA7-1***	7	12.21	Marker401588	5.01	0.40	3.28	BLUP	Significant	*qFA7_6*	Li et al. 2017	*Glyma07g03490* (*PLD*)	Kuppusamy et al. 2014
***qLA7-2***	7	40.33	Marker297491	8.47; 3.56	−0.77 −0.33	6.06; 2.29;	NJ2015; BLUP	Significant	*qLA7_2*	Li et al. 2017	*Glyma07g08740* (*PLD*)	Kuppusamy et al. 2014
***qLA7-4***	7	79.68; 83.71	Marker274074; Marker347920	4.00; 4.20	0.53; 0.69	2.88; 3.41	NJ2015; WH2014	Significant			*Glyma07g17720* (*GPAT6*)	Misra et al. (2017)
*qLA8-2*	8	39.09	Marker791165~Marker718573	3.37	−0.57	2.08	EZ2015	Suggestive	*qSA8_1*	Li et al. (2017)		
***qLA9-1***	9	151.54	Marker468763	3.42	−0.32	2.11	BLUP	Suggestive	*qPA9_6*	Li et al. 2017	*Glyma09g39911* (*DGD*)	Kelly et al. 2003
*qLA11-1*	11	47.19	Marker584683	2.59	−0.50	1.79	WH2014	Suggestive	*seed linoleic 1-5*	Diers and Shoemaker (1992)		
***qLA11-2***	11	79.33	Marker575184	3.26	0.31	1.94	BLUP	Suggestive			*Glyma11g36460*	Salminen et al. 2016
***qLA12-1***	12	59.46	Marker2734590	7.48	−0.71	5.12	NJ2015	Significant	*qFA12_2*	Li et al. 2017	*Glyma12g08010* (*KCS*)	Todd et al. 1999; Lee et al. 2010
*qLA12-2*	12	65.93	Marker2675339	5.75	−0.41	3.48	BLUP	Significant			*Glyma12g07230*	
***qLA13-1***	13	65.95	Marker2850221	4.28	−0.54	3.00	NJ2015	Significant	*seed linoleic 6-3*	Bachlava et al. (2009)	*Glyma13g16790* (*PDAT*)	Dahlqvist et al. (2000); Eskandari et al. 2013
*qLA13-2*	13	90.96	Marker2775509	14.39;9.69	−1.29; −0.55	10.70; 6.24	EZ2015; BLUP	Significant	*qOil-13-1*	Cao et al. (2017)		
*qLA13-3*	13	98.49	Marker2761097	4.03	−0.53	2.85	NJ2015	Significant	*qFA13_3*	Li et al. 2017		
***qLA13-5***	13	156.00	Marker2850173	3.02; 4.53	0.56; 0.37;	2.03; 2.74	EZ2015; BLUP	Significant	*seed linoleic 7-3*	Kim et al. (2010)	*Glyma13g42310* (*LOX*)*; Glyma13g42320* (*LOX*)	Radmark et al. 2007
***qLA14-1***	14	26.12; 26.81; 28.59;	Marker1690474; Marker1658604 Marker1626134	5.07 9.27 11.86 4.98	−0.73 −0.52 −0.90 −0.73	3.84 5.62 8.26 3.42	WH2014; EZ2015 NJ2015; BLUP	Significant	*qFA14_4*	Li et al. 2017	*Glyma14g08400* (*LPAT*)	Misra et al. 2014.
***qLA16-1***	16	115.60	Marker2543407	2.69	0.52	2.00	WH2014	Suggestive	*qPA16_2*	Li et al. 2017	*Glyma16g01070* (*LOX*)	Radmark et al. 2007
***qLA19-2***	19	43.08; 44.28	Marker1495349; Marker1590191	3.27; 4.33	−0.46 −0.36	2.21 2.65	NJ2015; BLUP	Significant	*qLA19_4*	Li et al. 2017		
*qLA20-1*	20	21.77	Marker1429985	2.51	−0.49	1.76	WH2014	Suggestive	*seed linoleic 6-8*	Bachlava et al. (2009); Fan et al. (2015)		
***qLA20-2***	20	37.30	Marker1439858	2.86	−0.29	1.77	BLUP	Suggestive	*seed linoleic 6-8*	Bachlava et al. (2009); Fan et al. (2015)	*Glyma20g25640* (*PAP*)*; Glyma20g25650* (*PAP*)	Millar et al. 2000

^a^ Proportion of total phenotypic variance explained by single QTL

^b^ Environment for the detected QTL

^c^ Genes around the detected QTLs in this study. The same QTLs using the linkage maps of including and excluding very significantly distorted markers are marked by bold type

#### Effect of marker segregation distortion on QTL mapping

If the linkage maps excluding very significantly distorted markers (case II) were used to conduct QTL mapping, the LOD score thresholds at the 0.05 probability level were 3.65, 3.70, 3.67, and 3.61, respectively, for the datasets I to IV. A total of 15 significant QTLs were detected. Among these 15 QTLs, 2, 4, 6 and 9 were found to be associated with linoleic acid content, respectively, for the datasets I to IV; 11 were previously reported; 13 were around the lipid metabolism related genes in Zhang et al. 2016) (Fig S7, Tables S4). In addition, a total of 5 suggestive QTLs were identified, 2, 3, 4 and 5 were found to be associated with linoleic acid content, respectively, for the datasets I to IV; 2 were previously reported; 3 were around the lipid metabolism related genes in Zhang et al. (2016) (Fig S7, Table S4). In summary, a total of 20 QTLs were identified, 13 were previously reported QTLs, and 16 were around the lipid metabolism related genes (Fig. S7, Table S4). As described above, suggestive QTLs were also confirmed by previously reported QTLs and seed oil biosynthesis genes in cases I and II. Thus, we compared the results of all the significant and suggestive QTLs in the above two cases. As a result, most (14) QTLs were the same. Of course, some differences were also identified. Ten QTLs (*qLA2-1*, *qLA4-1*, *qLA5-1*, *qLA7-3*, *qLA8-2*, *qLA11-1*, *qLA12-2*, *qLA13-2*, *qLA13-3* and *qLA20-1*) were detected only in case I, while six QTLs (*qLA1-1*, *qLA6-1*, *qLA8-1*, *qLA10-1*, *qLA13-4* and *qLA19-2*) were identified only in case II. Among these different QTLs, 7 and 2 were previously reported QTLs, respectively, in cases I and II; there were 4 different lipid metabolism related genes in each of these two cases. Thus, more information was obtained from case I (Table 4).

**Table 4:** Comparison of the soybean linoleic acid content QTLs using linkage maps generated by either including or excluding very significantly distorted markers

Case	QTLs from linkage maps that include very significantly distorted markers			QTLs from linkage maps that exclude very significantly distorted markers		
	No. of detected QTLs	No. of previously reported QTLs	No. of lipid metabolism related genes around detected QTLs	No. of detected QTLs	No. of previously reported QTLs	No. of lipid metabolism related genes around detected QTLs
Wuhan 2014	8 (3^a^)	6 (3)	5 (3)	4 (3)	3 (3)	3 (3)
Ezhou 2015	4 (2)	4 (2)	2 (2)	7 (2)	4 (2)	7 (2)
Nanjing 2015	10 (7)	8 (5)	7 (6)	10 (7)	8 (5)	8 (6)
BLUP	12 (10)	10 (8)	9 (8)	14 (10)	12 (8)	12 (8)
Total	24 (14)	18 (11)	16 (12)	20 (14)	13 (11)	16 (12)

^a^The number of the same QTLs identified with the linkage maps of both including and excluding very significantly distorted markers were listed in the bracket, and the same is true for the number of previously reported QTLs and the number of lipid metabolism related genes around QTLs in this study. The QTLs include significant and suggestive ones

## Discussion

Although some high-density genetic linkage maps have been previously reported in soybean (Li et al. 2014; Qi et al. 2014; Song et al. 2016; Li et al. 2017), the genetic linkage maps in this study have advantages in two aspects. On one hand, we included all the very significantly distorted markers in the current linkage maps and investigated the effect of these distorted markers on linkage map construction, especially on the order of markers on the linkage maps. This has not been reported in previous studies. In reality, marker segregation distortion is a natural phenomenon (Lyttle 1991). In previous studies, very significantly distorted markers were simply discarded in the construction of linkage maps. Clearly, this treatment results in the loss of marker information. In the present study, the number of linkage maps derived from all the normal and distorted markers is exactly same as the number of chromosomes in soybean, and all the 11,846 markers are grouped consistently in all the OC, RC and all the RILs. If we exclude all the very significantly distorted markers, the total genetic distance and marker density decrease, and the average marker-distance increases, specifically, the collinearity of soybean reference genome with most linkage maps is slightly reduced. Although relatively high collinearity in case II was observed on chromosomes 2, 11 and 17, the numbers of markers on these chromosomes are very limited. Thus, the current linkage maps including very significantly distorted markers are better than those excluding the very significantly distorted markers. On the other hand, all the RILs from orthogonal and reciprocal crosses are used to construct the current linkage maps. In this situation, the number of distorted markers in all the RILs (1999) is much less than those in OC (2306) or RC (2431) RILs, especially, on chromosomes 2, 13 and 19, and the quality of linkage maps can be improved. Therefore, all the markers in the bi-parental segregation population from orthogonal and reciprocal crosses should be used to construct linkage maps in soybean.

Marker segregation distortion may be due to various reasons (Lyttle 1991). In this study, the numbers of distorted markers on chromosomes 2, 6, 13, 18, and 19 in all the RILs are significantly less than the total number of distorted markers in OC and RC RILs. In the two sub-populations, the distorted markers are biased towards female parental genotypes on chromosomes 2, 18 and 19 and towards male parental genotypes on chromosome 6. On chromosome 13, these distorted markers in OC RILs are biased towards female parental genotypes, while 72 (15.6%) and 389 (84.4%) distorted markers in RC RILs are biased towards male and female parental genotypes, respectively. Obviously, the distortion may be caused by gametic selection. This hypothesis is partly supported by the presence of male sterile genes on chromosomes 2 and 13 (Jin et al. 1998; Yang et al. 2014). Based on the above results, we doubt that there are female and male sterile genes, respectively, in chromosomes 6 and 19. On chromosome 17, however, there are 1130, 15 and 1097 distorted markers, respectively, in OC, RC and all the RILs. These distorted markers are biased toward female parental genotypes in OC RILs and toward male parental genotypes in RC RILs, and the reason for marker segregation distortion may be different from those in the above situations.

The current linkage maps have been validated in two aspects. On one hand, all the 11,846 markers are exactly clustered into 20 chromosomes in OC, RC and all the RILs, and the number of markers for each chromosome in the above three populations is exactly the same. The collinearities of the soybean reference genome with each linkage map are relatively high. The recombination fractions in the terminals for each chromosome were significantly higher than those in the middle intervals, which is consistent with results from previous studies. On the other hand, the results of mapping QTL for linoleic acid content in soybean in this study also validate the accuracy of the linkage maps. This is because, among 24 QTLs identified in this study, 18 are previously reported QTLs, and 16 are around lipid metabolism related genes (Zhang et al. 2016) (Table S4).

As compared with the results of excluding distorted markers, inclusion of distorted markers increases the accuracy of grouping all the markers on chromosomes. The reasons are as follows. First, the numbers of distorted markers in chromosomes 2, 6, 13, 18, and 19 in all the RILs are significantly fewer than those in OC or RC RILs. Use of fewer distorted markers in all the RILs decreases the impact of distorted marker on linkage map construction (Zhu et al. 2007; Xie et al. 2014), and the quality of the linkage maps increases. Second, most distorted markers are clustered. Once these distorted markers are excluded from the construction of linkage maps, a marker-gap in the linkage map exists, for example chromosome 17. In addition, distorted markers increase the consistency of linkage maps with genome and genome coverage. As described in Table S2 in RC RILs, the Spearman correlation coefficient from chromosome 2 by including distorted markers (0.8737) was higher than that for excluding distorted markers (0.8365).

## Conclusion

A total of 11,846 SLAF markers in all the RILs were mapped onto 20 chromosomes with an average marker-interval of 0.21 cM. A total of 1513 (12.77%) very significantly distorted markers increased the accuracy of grouping markers on their corresponding chromosomes, the consistency of linkage maps with genome and genome coverage. Less very significantly distorted markers in all the RILs than in OC and RC RILs improved the quality of linkage maps. The relatively high collinearity of linkage maps with the reference genome, and previously reported QTLs and lipid metabolism related genes around 24 seed linoleic acid content QTLs detected in this study validated the quality of the linkage maps.

## Supplementary information

Supplementary material
