# Supplementary material for: Effect of marker segregation distortion on high density linkage map construction and QTL mapping in Soybean (Glycine max L.)
Source: Heredity (Edinb). 2019 May 31;123(5):579–92. doi: 10.1038/s41437-019-0238-7 (PMC6972858; doi:10.1038/s41437-019-0238-7)
Supplement: Supplementary file 1 — Supplementary material [file 41437_2019_238_MOESM1_ESM.pdf]

Effect of Marker Segregation Distortion on High Density Linkage Map Construction and QTL Mapping in Soybean (*Glycine max* L.)

Jian-Fang Zuo[1], Yuan Niu[2], Peng Cheng[1], Jian-Ying Feng[3], Shi-Feng Han[3], Ying-Hao Zhang[1], Guoping Shu[4], Yibo Wang[4], and Yuan-Ming Zhang[1 *]

1 Crop Information Center, College of Plant Science and Technology, Huazhong Agricultural University, Wuhan 430070, China

2 College of Life Sciences and Food Engineering, Huaiyin Institute of Technology, Huaian 223003, China

3 State Key Laboratory of Crop Genetics and Germplasm Enhancement, Nanjing Agricultural University, Nanjing 210095, China

4 Center of Molecular Breeding and Biotechnology, Beijing Lantron Seed Corp., Beijing 100081, China

*** Correspondence:**

Yuan-Ming Zhang

soyzhang@mail.hzau.edu.cn

SUPPLEMENTARY MATERIAL

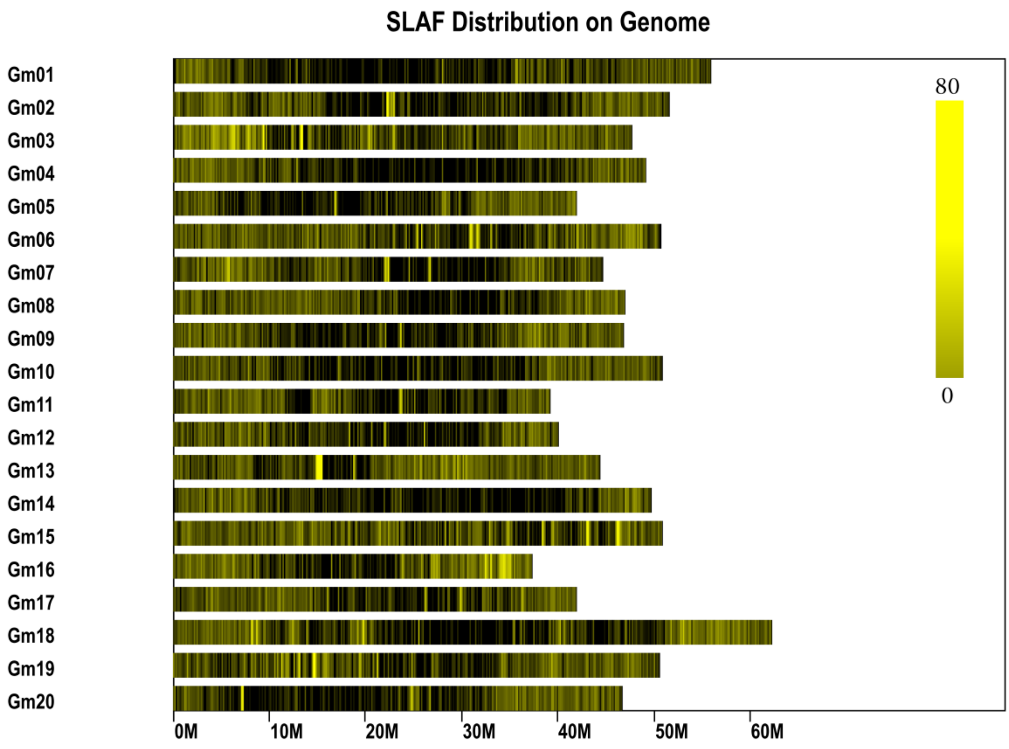

Figure S1. The SLAF markers distributed on the soybean genome

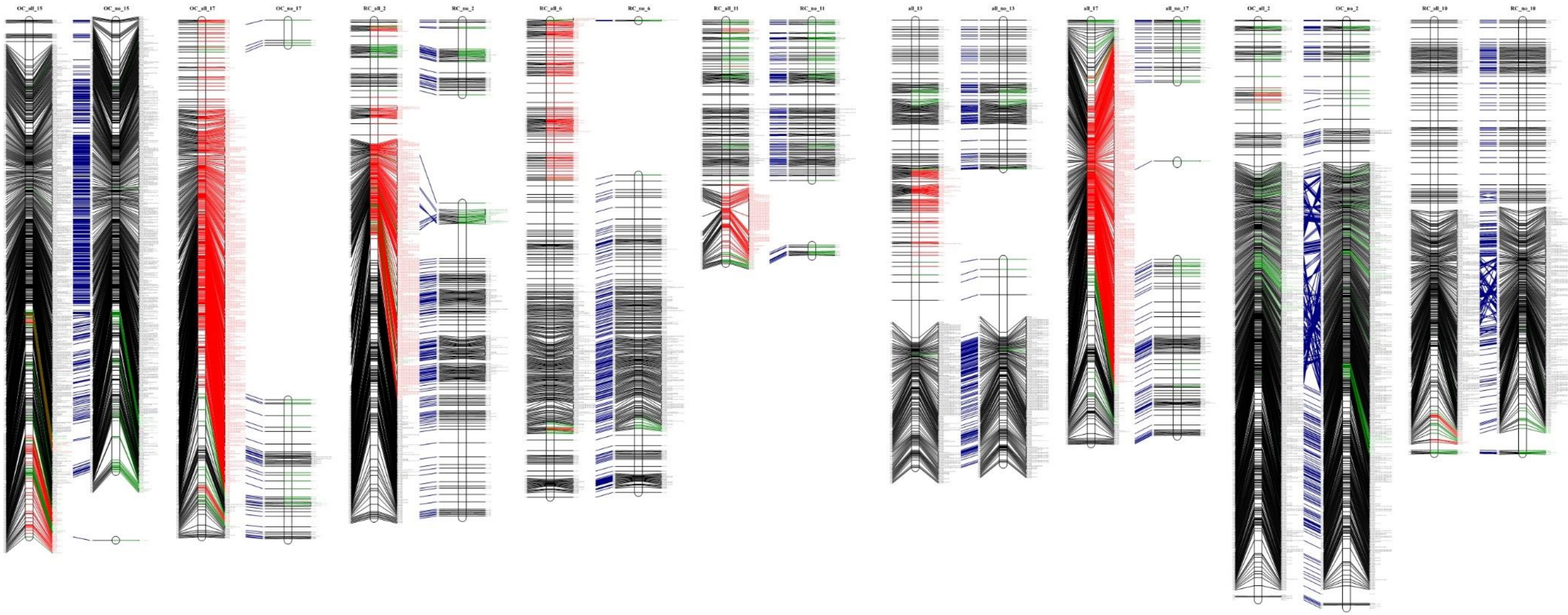

Figure S2. The effect of very significantly distorted markers on the construction of linkage maps. The significantly distorted markers at the 0.05 and 0.01 probability levels are marked by blue and red colors, respectively. OC: orthogonal cross; RC: reciprocal cross; all: including very significantly distorted markers in the linkage maps; no: excluding very significantly distorted markers in the linkage map; number: chromosome code.

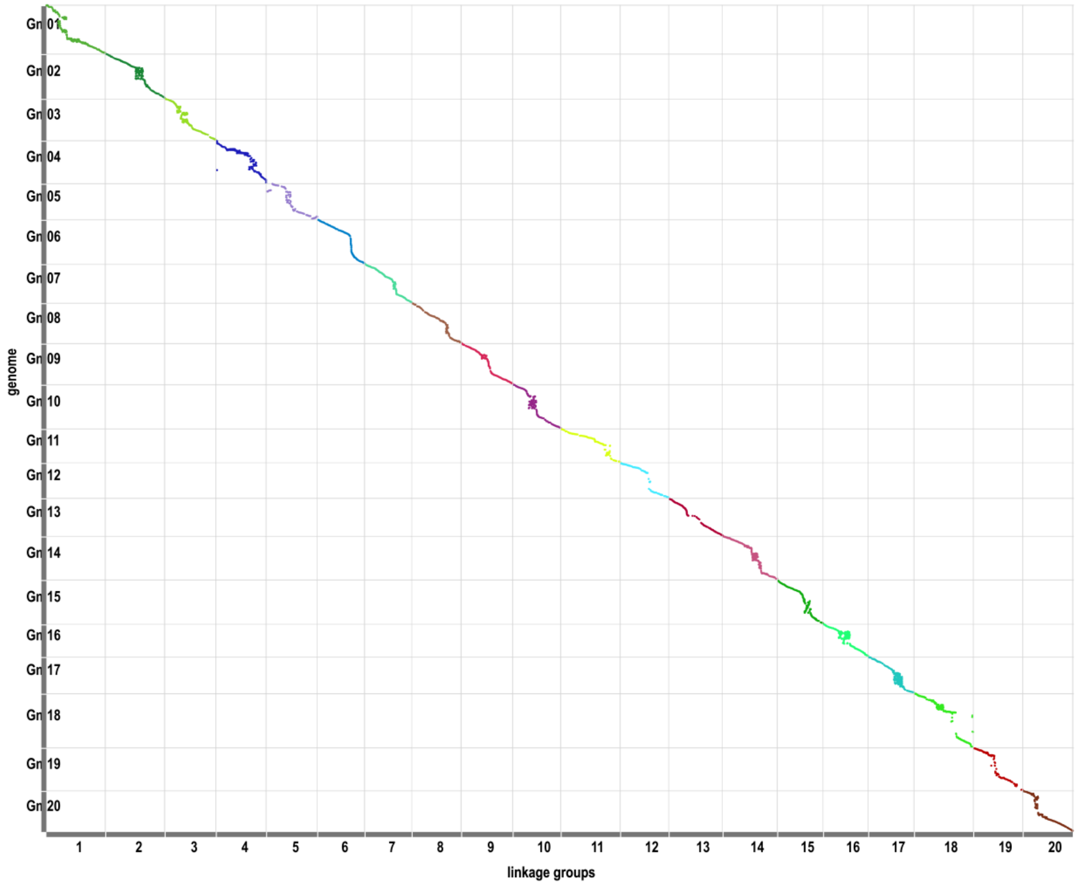

Figure S3. The collinearity of linkage maps with soybean reference genome

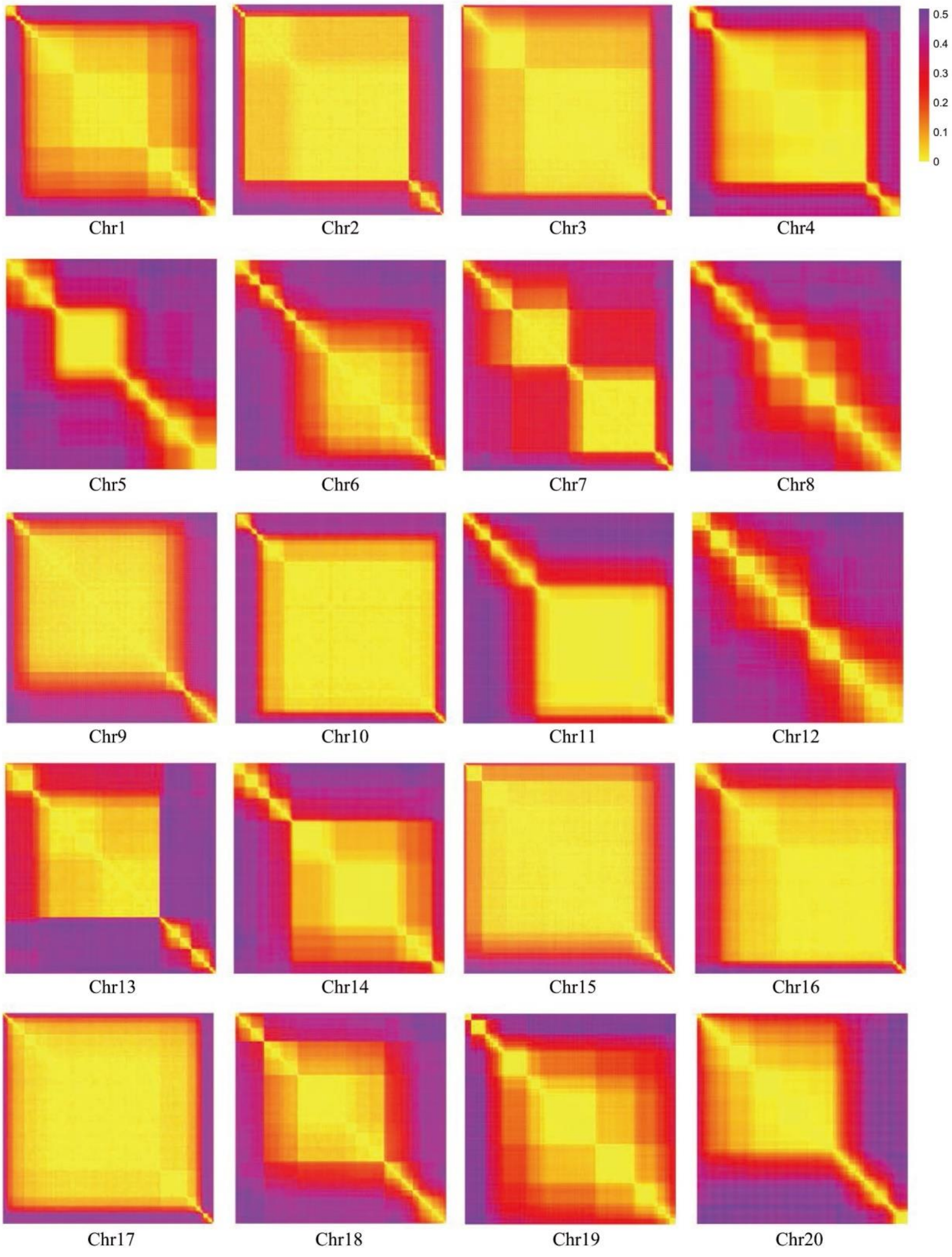

Figure S4. The heat map indicated by the matrices of pair-wise recombination fractions of SLAF markers. The X (horizontal) and Y (vertical) axes indicate the markers on each chromosome, and the diagonal represents the recombination fraction of 0.0 for the same markers, and the color of cell shows the size of recombination fraction of two markers.

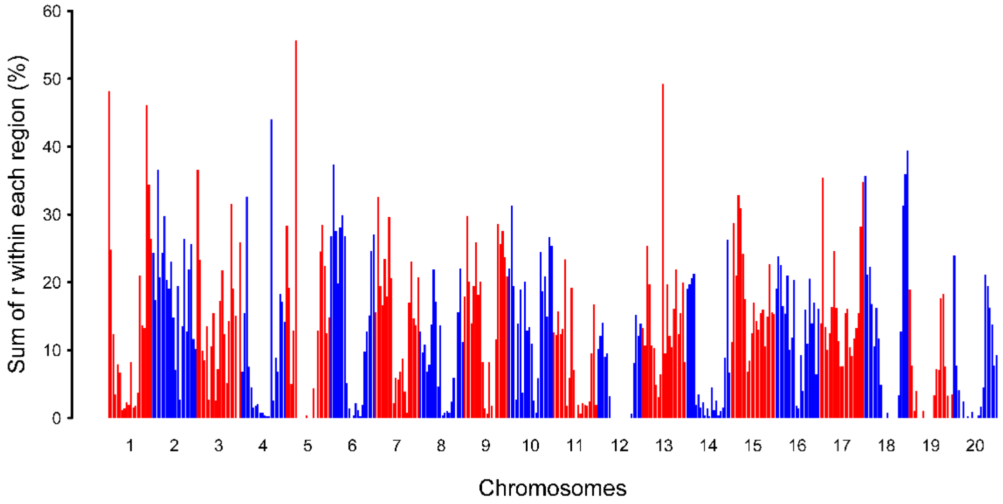

Figure S5. Interval recombinant fractions on 20 soybean chromosomes. The red and blue lines represent odd and even numbers of chromosomes, respectively**.**

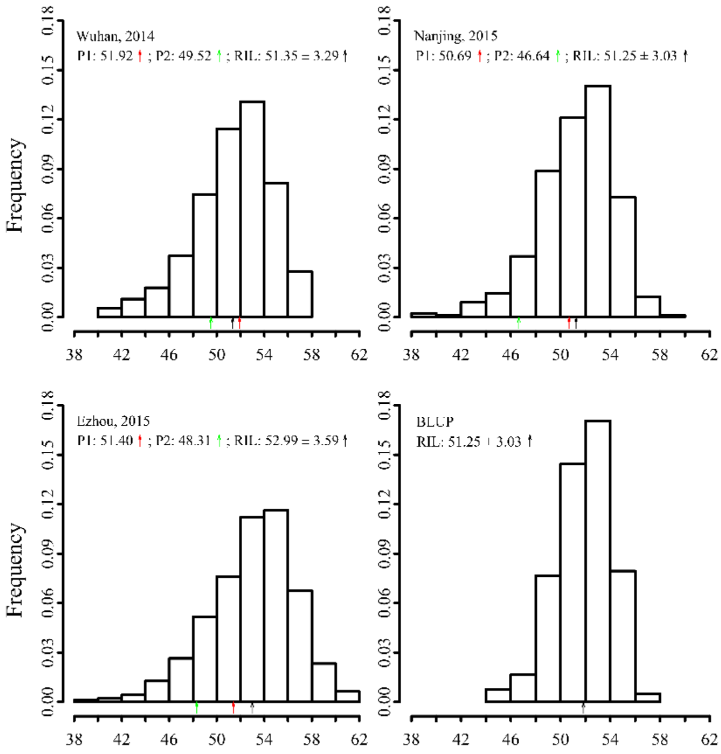

Figure S6. Frequency distributions of soybean seed linoleic acid content in Wuhan (2014), Ezhou (2015), Nanjing (2015), and the BLUP values. The averages for seed linoleic acid contents in P_1, P_2 and RILs are marked by red, green and black arrows, respectively**.**

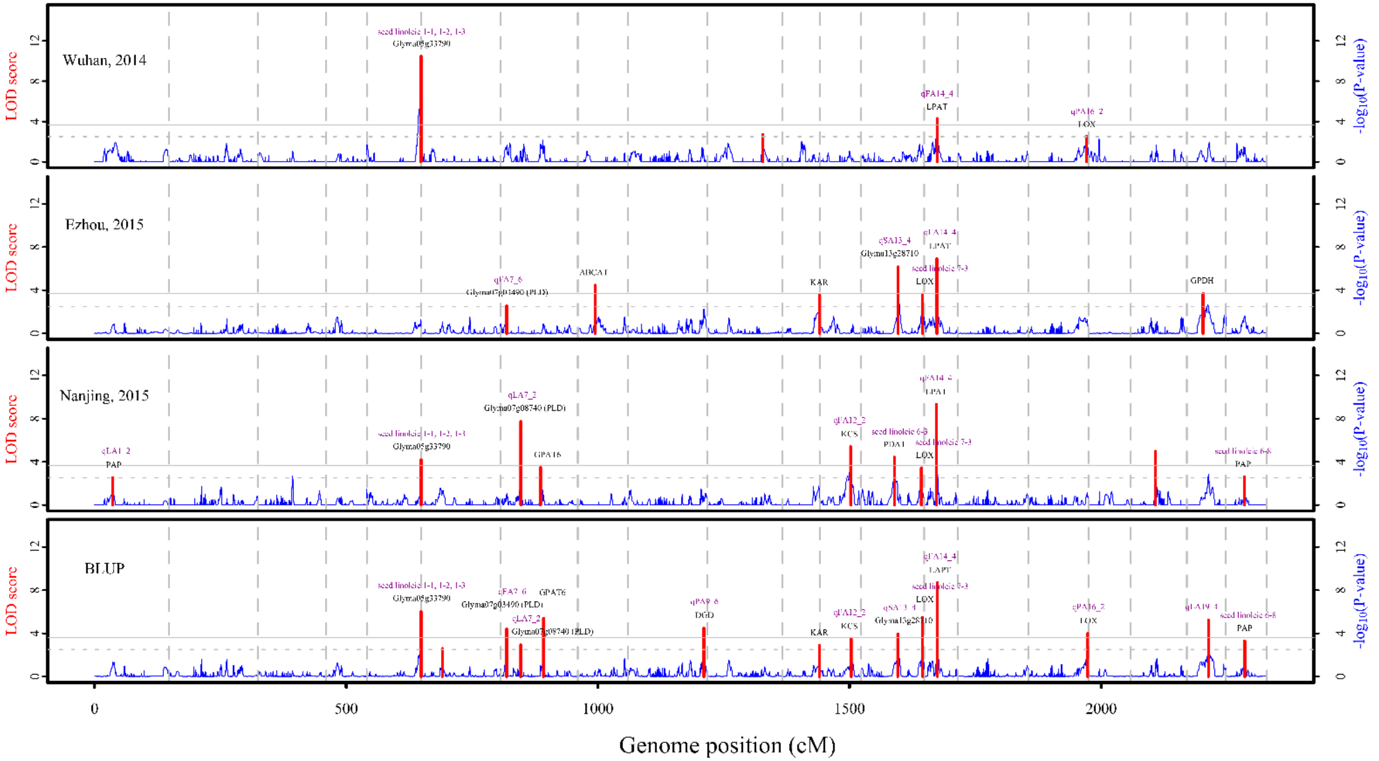

Figure S7. QTLs for soybean seed linoleic acid content in Wuhan, Ezhou, Nanjing and the BLUP values from linkage maps that exclude very significantly distorted markers using QTL.gCIMapping.GUI. The $-\log_{10}$(P-value) and LOD scores are indicated respectively by the blue and red lines. Grey solid and dotted horizontal lines are the LOD score thresholds respectively for significant and suggestive QTLs. Previously reported QTLs and lipid metabolism related genes are marked respectively by dark magenta and black colors.

Table S1. Comparison of the number of markers, total (Total) and average (Average) genetic distances for linkage groups either including or excluding very significant markers with P-values ≤ 0.01 in orthogonal cross (OC), reciprocal cross (RC) and all the RILs

Chr	Each linkage map of including all markers							Each linkage map of excluding highly significant distorted markers with the P-values ≤ 0.01								
	No. of markers	All the RILs		OC RILs		RC RILs		All the RILs			OC RILs			RC RILs		
		Total	Average	Total	Average	Total	Average	No. of markers	Total	Average	No. of markers	Total	Average	No. of markers	Total	Average
1	507	150.48	0.30	147.36	0.29	158.33	0.31	479	148.02	0.31	478	144.42	0.30	507	158.33	0.31
2	1116	176.26	0.16	**206.21**	0.18	177.20	0.16	1116	176.26	0.16	1107	209.12	0.19	**225+34**	**111.33+26.50**	0.53
3	917	137.01	0.15	146.49	0.16	149.49	0.16	899	136.10	0.15	916	146.77	0.16	915	149.49	0.16
4	277	80.78	0.29	79.27	0.29	86.63	0.31	277	80.78	0.29	277	79.27	0.29	277	86.63	0.31
5	152	107.95	0.71	107.96	0.71	107.92	0.71	152	107.95	0.71	143	102.49	0.72	152	107.92	0.71
6	466	158.82	0.34	164.10	0.35	169.70	0.36	453	157.60	0.35	466	164.10	0.35	**359+4**	**112.92+0.19**	0.31
7	637	153.64	0.24	157.59	0.25	177.66	0.28	637	153.64	0.24	634	159.13	0.25	627	176.44	0.28
8	229	100.04	0.44	101.29	0.44	104.72	0.46	229	100.04	0.44	202	100.73	0.50	228	104.73	0.46
9	917	158.93	0.17	159.14	0.17	196.18	0.21	895	157.02	0.18	897	158.89	0.18	917	194.18	0.21
10	669	149.87	0.22	155.36	0.23	154.13	0.23	668	149.77	0.22	663	155.21	0.23	659	154.20	0.23
11	354	80.02	0.23	79.70	0.23	85.53	0.24	**167**	73.89	0.44	354	79.70	0.23	**117+9**	**56.97+3.55**	0.48
12	163	82.72	0.51	74.00	0.45	85.45	0.52	160	82.50	0.52	149	71.55	0.48	154	85.81	0.56
13	646	159.15	0.25	185.81	0.29	163.71	0.25	**477+93**	**72.99+52.86**	0.22	637	186.12	0.29	**241**	158.79	0.66
14	499	67.07	0.13	68.13	0.14	79.26	0.16	499	67.07	0.13	490	68.15	0.14	499	79.26	0.16
15	1327	147.50	0.11	183.80	0.14	159.18	0.12	1314	145.83	0.11	**1259+2**	**160.57+0.00**	0.13	1318	158.20	0.12
16	670	119.53	0.18	138.46	0.21	118.09	0.18	670	119.53	0.18	670	138.46	0.21	670	118.09	0.18
17	**1168**	150.68	0.13	183.79	0.16	154.71	0.13	**72+24+2**	**62.58+22.03+0.00**	0.86	**57+4**	**49.47+8.90**	0.96	1168	154.71	0.13
18	**610**	136.60	0.22	139.83	0.23	154.69	0.25	**530**	**111.14**	0.21	**370**	**128.74**	0.35	**530**	**126.86**	0.24
19	236	77.81	0.33	77.81	0.33	77.27	0.33	236	77.81	0.33	235	77.58	0.33	144	86.91	0.6
20	286	81.01	0.28	75.81	0.27	83.40	0.29	284	81.33	0.29	280	75.35	0.27	270	83.09	0.31
Total	11846	2475.86	0.21	2631.89	0.22	2643.24	0.22	10333	2336.74	0.23	10290	2464.72	0.24	10024	2495.10	0.25

Table S2. Comparison of correlation coefficients between soybean reference genome and linkage maps including (Including) or excluding (Excluding) highly significant markers with the P-values ≤ 0.01 in orthogonal cross (OC), reciprocal cross (RC) and all the RILs

Chromosome	OC RILs			RC RILs			All the RILs		
	Including (A)	Excluding (B)	Difference (B−A)	Including (A)	Excluding (B)	Difference (B−A)	Including (A)	Excluding (B)	Difference (B−A)
1	0.9831	0.9799	-0.0033	0.9857	0.9857	0.0000	0.9962	0.9955	-0.0007
2	0.8737	0.8365	-0.0371	0.8848	0.9681	**0.0833**	0.8705	0.8705	0.0000
3	0.9271	0.9269	-0.0002	0.8773	0.8765	-0.0008	0.9372	0.9333	-0.0038
4	0.9387	0.9387	0.0000	0.9392	0.9392	0.0000	0.9406	0.9406	0.0000
5	0.9356	0.9324	-0.0032	0.9338	0.9338	0.0000	0.9339	0.9339	0.0000
6	0.9995	0.9995	0.0000	0.9987	0.9972	-0.0014	0.9998	0.9998	0.0000
7	0.9545	0.9538	-0.0006	0.9534	0.9512	-0.0023	0.9645	0.9645	0.0000
8	0.9991	0.9988	-0.0003	0.9990	0.9990	0.0000	0.9991	0.9991	0.0000
9	0.9551	0.9521	-0.0031	0.8997	0.8997	0.0000	0.9850	0.9839	-0.0011
10	0.6557	0.6463	-0.0094	0.7813	0.7884	0.0071	0.8214	0.8206	-0.0008
11	0.8208	0.8208	0.0000	0.8157	0.6254	-0.1903	0.8414	0.9963	**0.1549**
12	0.9988	0.9986	-0.0002	0.9992	0.9992	0.0000	0.9992	0.9993	0.0001
13	0.9897	0.9893	-0.0004	0.9889	0.9998	0.0109	0.9999	0.8743	-0.1256
14	0.9858	0.9850	-0.0008	0.9879	0.9879	0.0000	0.9883	0.9883	0.0000
15	0.8918	0.8644	-0.0274	0.8488	0.8457	-0.0031	0.9431	0.9414	-0.0017
16	0.8837	0.8837	0.0000	0.8669	0.8669	0.0000	0.8950	0.8950	0.0000
17	0.8889	0.9969	**0.1080**	0.8095	0.8095	0.0000	0.9550	0.9486	-0.0063
18	0.9901	0.9996	0.0095	0.9933	0.9898	-0.0035	0.9926	0.9887	-0.0039
19	0.9941	0.9940	-0.0001	0.9950	0.9902	-0.0048	0.9961	0.9961	0.0000
20	0.9964	0.9962	-0.0002	0.9953	0.9945	-0.0007	0.9968	0.9967	-0.0001

Table S3. Phenotypic characteristics for soybean seed linoleic acid content (%) in all the RILs and their parents

Case	Parents		All the RILs						
	LSZZH (%)	NN493-1 (%)	Mean (%)	Minimum (%)	Maximum (%)	SD (%)[a]	CV (%)[b]	Skewness	Kurtosis
Wuhan, 2014	51.92	49.52	51.35	40.64	57.70	3.29	6.41	-0.69	0.43
Ezhou, 2015	51.4	48.31	52.99	39.47	61.86	3.59	6.77	-0.52	0.35
Nanjing, 2015	50.69	46.64	51.25	39.87	58.23	3.03	5.92	-0.78	0.95
BLUP			51.84	44.14	56.26	2.20	4.24	-0.57	0.20

[a] standard deviation; [b] coefficient of variation

Table S4. QTLs detected for linoleic acid content of soybean seed in all the three environments using the QTL.gCIMapping.GUI program

QTL	Chr	QTLs detected							Previously reported QTLs		Lipid metabolism related genes around detected QTLs	
		Position (cM)	Marker intervals	LOD	Effect	r^2 (%) [a]	Environment [b]	Type	QTL	Reference	Gene [c]	Reference
**QTLs detected in this study using linkage maps including very highly significantly distorted markers**												
qLA2-1	2	111.81	Marker1183683	2.85	-0.54	2.11	WH2014	Suggestive	*qFA2*	Li et al. 2017	*Glyma02g13700*	
qLA4-1	4	22.26	Marker2230222	3.28	0.31	1.99	BLUP	Suggestive			*Glyma04g37420* (*FATB*)	Salas & Ohlrogge 2002;Ozseyhan et al. 2018
qLA5-1	5	7.97	Marker2204980	2.74	0.41	1.75	NJ2015	Suggestive	*qOA5_3*	Li et al. 2017	*Glyma05g07880* (*OBO*)	Wang et al. 2008
qLA5-2	5	107.75;<br>107.95	Marker2104599;<br>Marker2161702	6.47;<br>4.24;<br>10.72	0.43;<br>1.07;<br>0.52	3.84;<br>8.25;<br>2.77	BLUP;<br>WH2014;<br>NJ2015	Significant	*seed linoleic 1-1, 1-2, 1-3*	Diers & Shoemaker (1992);<br>Li et al. (2017); Cao et al. (2017)	*Glyma05g33790*	Chen et al. 2018
qLA7-1	7	12.21	Marker401588	5.01	0.40	3.28	BLUP	Significant	*qFA7_6*	Li et al. 2017	*Glyma07g03490* (*PLD*)	Kuppusamy et al. 2014
qLA7-2	7	40.33	Marker297491	8.47;<br>3.56	-0.77<br>-0.33	6.06;<br>2.29;	NJ2015;<br>BLUP	Significant	*qLA7_2*	Li et al. 2017	*Glyma07g08740* (*PLD*)	Kuppusamy et al. 2014
qLA7-3	7	46.63	Marker336577	3.04	-0.58	2.41	WH2014	Suggestive				
qLA7-4	7	79.68;<br>83.71	Marker274074;<br>Marker347920	4.00;<br>4.20	0.53;<br>0.69	2.88;<br>3.41	NJ2015;<br>WH2014	Significant			*Glyma07g17720* (*GPAT6*)	Misra and Khan. 2017
qLA8-2	8	39.09	Marker791165~Marker718573	3.37	-0.57	2.08	EZ2015	Suggestive	*qSA8_1*	Li et al. (2017)		
qLA9-1	9	151.54	Marker468763	3.42	-0.32	2.11	BLUP	Suggestive	*qPA9_6*	Li et al. 2017	*Glyma09g39911* (*DGD*)	Kelly et al. 2003
qLA11-1	11	47.19	Marker584683	2.59	-0.50	1.79	WH2014	Suggestive	*seed linoleic 1-5*	Diers & Shoemaker (1992)		
qLA11-2	11	79.33	Marker575184	3.26	0.31	1.94	BLUP	Suggestive			*Glyma11g36460*	Salminen et al. 2016
qLA12-1	12	59.46	Marker2734590	7.48	-0.71	5.12	NJ2015	Significant	*qFA12_2*	Li et al. 2017	*Glyma12g08010* (*KCS*)	Todd et al. 1999; Lee et al. 2010
qLA12-2	12	65.93	Marker2675339	5.75	-0.41	3.48	BLUP	Significant			*Glyma12g07230*	

qLA13-1	13	65.95	Marker2850221	4.28	-0.54	3.00	NJ2015	Significant	*seed linoleic 6-3*	Bachlava et al. (2009)	*Glyma13g16790* (*PDAT*)	Dahlqvist et al. (2000); Eskandari et al. 2013
qLA13-2	13	90.96	Marker2775509	14.39; 9.69	-1.29; -0.55	10.70; 6.24	EZ2015; BLUP	Significant	*qOil-13-1*	Cao et al. (2017)		
qLA13-3	13	98.49	Marker2761097	4.03	-0.53	2.85	NJ2015	Significant	*qFA13_3*	Li et al. 2017		
qLA13-5	13	156.00	Marker2850173	3.02; 4.53	0.56; 0.37;	2.03; 2.74	EZ2015; BLUP	Significant	*seed linoleic 7-3*	Kim et al. (2010)	*Glyma13g42310* (*LOX*); *Glyma13g42320* (*LOX*)	Radmark et al. 2007
qLA14-1	14	26.12; 26.81; 28.59	Marker1690474; Marker1658604; Marker1626134	5.07; 9.27; 11.86; 4.98	-0.73; -0.52; -0.90; -0.73	3.84; 5.62; 8.26; 3.42	WH2014; EZ2015; NJ2015; BLUP	Significant	*qFA14_4*	Li et al. 2017	*Glyma14g08400* (*LPAT*)	Misra et al. 2014.
qLA16-1	16	115.60	Marker2543407	2.69	0.52	2.00	WH2014	Suggestive	*qPA16_2*	Li et al. 2017	*Glyma16g01070* (*LOX*)	Radmark et al. 2007
qLA18-1	18	48.86	Marker950775	6.45	0.66	4.45	NJ2015	Significant				
qLA19-2	19	43.08; 44.28	Marker1495349; Marker1590191	3.27; 4.33	-0.46; -0.36	2.21; 2.65	NJ2015; BLUP	Significant	*qLA19_4*	Li et al. 2017		
qLA20-1	20	21.77	Marker1429985	2.51	-0.49	1.76	WH2014	Suggestive	*seed linoleic 6-8*	Bachlava et al. (2009); Fan et al. (2015)		
qLA20-2	20	37.30	Marker1439858	2.86	-0.29	1.77	BLUP	Suggestive	*seed linoleic 6-8*	Bachlava et al. (2009); Fan et al. (2015)	*Glyma20g25640* (*PAP*); *Glyma20g25650* (*PAP*)	Millar et al. 2000
**QTLs detected in this study using linkage maps excluding very highly significantly distorted markers**												
qLA1-1	1	36.17	Marker1774646	2.53	-0.44	1.59	NJ2015	Suggestive	*qLA1 _2*	Li et al. 2017	*Glyma01g37710* (*PAP*)	Millar et al. 2000
qLA5-2	5	107.75; 107.95	Marker2104599; Marker2161702	10.47; 6.00; 4.17	1.09; 0.43; 0.53 9	9.78; 2.78; 2.27	WH2014; BLUP; NJ2015	Significant	*seed linoleic 1-1, 1-2, 1-3*	Diers & Shoemaker (1992); Cao et al. (2017); Li et al. (2017);	*Glyma05g33790*	Chen et al. 2018
qLA6-1	6	42.27	Marker2019009	2.59	-0.27	1.04	BLUP	Suggestive				
qLA7-1	7	12.21	Marker401588	2.54; 4.40	0.50; 0.38	1.63; 2.11	EZ2015; BLUP	Significant	*qFA7_6*	Li et al. 2017	*Glyma07g03490* (*PLD*)	Kuppusamy et al. 2014
qLA7-2	7	40.33	Marker297491	2.92; 7.73	-0.32; -0.77	1.52; 4.83	BLUP; NJ2015	Significant	*qLA7_2*	Li et al. 2017	*Glyma07g08740* (*PLD*)	Kuppusamy et al. 2014
qLA7-4	7	79.68; 85.48	Marker274074; Marker300464	3.47; 5.40	0.51; 0.38	2.07; 2.15	NJ2015; BLUP	Significant			*Glyma07g17720* (*GPAT6*)	Misra and Khan. 2017
qLA8-1	8	34.59	Marker741533	4.46	-0.67	2.91	EZ2015	Significant			*Glyma08g17110* (*ABCAT*)	*Jouhe et al. 2007*
qLA9-1	9	150.54	Marker507211	4.49	-0.37	2.06	BLUP	Significant	*qPA9_6*	Li et al. 2017	*Glyma09g39911* (*DGD*)	Kelly et al. 2003
qLA10-1	10	110.41	Marker1001597	2.72	-0.54	2.41	WH2014	Suggestive				

qLA11-2	11	73.20; 73.89	Marker639248; Marker615463	2.90; 3.54	0.28; 0.59	1.14; 2.30	BLUP; EZ2015	Suggestive			*Glyma11g37320* (*KAR*)	Wu and Xue. 2010
qLA12-1	12	61.51; 62.51	Marker2711916; Marker2729868	5.40; 3.49	-0.65; -0.34	3.47; 1.72	NJ2015; BLUP	Significant	*qFA12_2*	Li et al. 2017	*Glyma12g08010* (*KCS*)	Todd et al. 1999; Lee et al. 2010
qLA13-1	13	65.95	Marker2850221	4.42	-0.58	2.77	NJ2015	Significant	*seed linoleic 6-3*	Bachlava et al. (2009)	*Glyma13g16790* (*PDAT*)	Dahlqvist et al. (2000); Eskandari et al. 2013
qLA13-4	13	72.60; 73.20	Marker2856397; Marker2836308	3.93; 6.16	-0.36; -0.77	1.92; 3.88	BLUP; EZ2015	Significant	*qSA13_4*	Li et al. 2017	*Glyma13g28710*	*Li et al. 2012*
qLA13-5	13	119.35; 121.72	Marker2798590; Marker2824551	3.43; 3.64; 5.48	0.49; 0.63; 0.41	1.94; 2.63; 2.46	NJ2015; EZ2015; BLUP	Significant	*seed linoleic 7-3*	Kim et al. (2010)	*Glyma13g42310* (*LOX*); *Glyma13g42320* (*LOX*)	Radmark et al. 2007
qLA14-1	14	24.22; 25.32; 26.12	Marker1636277; Marker1651052; Marker1690474	9.31; 6.92; 4.29; 8.68	-0.85; -0.82; -0.69; -0.54	5.85; 4.42; 3.89; 4.38	WH2014; EZ2015; NJ2015; BLUP	Significant	*qFA14_4*	Li et al. 2017	*Glyma14g08400* (*LPAT*)	Misra et al. 2014.
qLA16-1	16	115.60; 118.10	Marker2543407; Marker2585858	2.53; 3.98	0.52; 0.37;	2.24; 2.05;	WH2014; BLUP	Significant	*qPA16_2*	Li et al. 2017	*Glyma16g01070* (*LOX*)	Radmark et al. 2007
qLA18-1	18	48.86	Marker950775	4.97	0.58	2.77	NJ2015	Significant				
qLA19-1	19	32.30	Marker1474585	3.70	-0.62	2.48	EZ2015	Significant			*Glyma19g31730* (*GPDH*)	*Kim et al. 2015*
qLA19-2	19	43.08	Marker1495349	5.21	-0.41	2.50	BLUP	Significant	*qLA19_4*	Li et al. 2017		
qLA20-2	20	36.56; 37.30	Marker1386115; Marker1439858	2.60; 3.30	-0.39; -0.33	1.23; 1.60	NJ2015; BLUP	Suggestive	*seed linoleic 6-8*	Bachlava et al. (2009); Fan et al. (2015)	*Glyma20g25640* (*PAP*); *Glyma20g25650* (*PAP*)	Millar et al. 2000

[a] Proportion of total phenotypic variance explained by single QTL; [b] Environment for the existence of significant QTL; [c] Genes around the detected QTLs in this study. The same QTLs using linkage maps of including and excluding very significantly distorted markers are marked by bold type.

References

1 Bachlava E, Dewey RE, Burton JW, Cardinal AJ (2009) Mapping and comparison of quantitative trait loci for oleic acid seed content in two segregating soybean populations. Crop Sci 49:433-442.

2 Cao Y, Li S, Wang Z, Chang F, Kong J, Gai J, et al. (2017) Identification of major quantitative trait loci for seed oil content in soybeans by combining linkage and genome-wide association mapping. Front Plant Sci 8:1222.

3 Chen W, Salari H, Taylor MC, Jost R, Berkowitz O, Barrow R, et al. (2018) NMT1 and NMT3 N-methyltransferase activity is critical to lipid homeostasis, morphogenesis, and reproduction. Plant Physiol 177(4):1605-1628.

4 Dahlqvist A, Ståhl U, Lenman M, Banas A, Lee M, Sandager L, et al. (2000) Phospholipid:diacylglycerol acyltransferase: An enzyme that catalyzes the acyl-CoA-independent formation of triacylglycerol in yeast and plants. Proc Natl Acad Sci USA 97:6487-6492.

5 Diers BW, Shoemaker RC (1992) Restriction fragment length polymorphism analysis of soybean fatty acid content. J Am Oil Chem Soc 69:1242-1244.

6 Eskandari M, Cober ER, Rajcan I (2013) Using the candidate gene approach for detecting genes underlying seed oil concentration and yield in soybean. Theor Appl Genet 126 (7):1839-1850.

7 Fan S, Li B, Yu F, Han F, Yan S, Wang L, et al. (2015)Analysis of additive and epistatic quantitative trait loci underlying fatty acid concentrations in soybean seeds across multiple environments. Euphytica 206:689-700.

8 Jouhet J, Maréchal E, Block MA (2007) Glycerolipid transfer for the building of membranes in plant cells. Prog Lipid Res. 46(1):37-55.

9 Kelly AA, Froehlich JE, Dörmann P (2003) Disruption of the two digalactosyldiacylglycerol synthase genes *DGD1* and *DGD2* in Arabidopsis reveals the existence of an additional enzyme of galactolipid synthesis. Plant Cell 15(11):2694-2706.

10 Kim HK, Kim YC, Kim ST, Son BG, Choi YW, Kang JS, et al. (2010) Analysis of quantitative trait loci (QTLs) for seed size and fatty acid composition using recombinant inbred lines in soybean. J Life Sci 20:1186-1192.

11 Kim HU, Lee KR, Jung SJ, Shin HA, Go YS, Suh MC, et al. (2015) Senescence-inducible LEC2 enhances triacylglycerol accumulation in leaves without negatively affecting plant growth. Plant Biotechnol J. 13(9):1346-1359.

12 Kuppusamy T, Giavalisco P, Arvidsson S, Sulpice R, Stitt M, Finnegan PM, et al. (2014) Lipid biosynthesis and protein concentration respond uniquely to phosphate supply during leaf development in highly-phosphorus-efficient harsh hakea. Plant Physiol 166(4):1891-1911.

13 Lee SB, Jung SJ, Go YS, Kim HU, Kim JK, Cho HJ, et al. (2010) Two Arabidopsis 3-ketoacyl CoA synthase genes, *KCS20* and *KCS2/DAISY*, are functionally redundant in cuticular wax and root suberin biosynthesis, but differentially controlled by osmotic stress. Plant J 60:462-475.

14 Li B, Fan S, Yu F, Chen Y, Zhang S, Han F, et al. (2017) High-resolution mapping of QTL for fatty acid composition in soybean using specific-locus amplified fragment sequencing. Theor Appl Genet 130:1467-1479.

15 Li W, Ling H, Zhang F, Yao HY, Sun XF, Tang KX (2012) Analysis of *Arabidopsis* genes encoding putative class III lipases. J Plant Biochem Biotechnol. 21(2):261-267.

16 Millar AA, Smith MA, Kunst L (2000) All fatty acids are not equal: discrimination in plant membrane lipids. Trends Plant Sci. 5(3):95-101

17 Misra A, Khan K, Niranjan A, Kumar V, Sane VA (2017) Heterologous expression of two GPATs from *Jatropha curcas* alters seed oil levels in transgenic *Arabidopsis thaliana*. Plant Sci. 263:79-88

18 Misra N, Panda PK, Parida BK (2014) Genome-wide identification and evolutionary analysis of algal *LPAT* genes involved in TAG biosynthesis using bioinformatic approaches. Mol Biol Rep 41:8319-8332.

19 Ozseyhan ME, Li P, Na G, Li Z, Wang C, Lu C (2018) Improved fatty acid profiles in seeds of *Camelina sativa* by artificial microRNA mediated FATB gene suppression. Biochem Biophys Res Commun 503:621-624.

20 Radmark O, Werz O, Steinhilber D, Samuelsson B (2007) 5-Lipoxygenase: regulation of expression and enzyme activity. Trends Biochem Sci 32:332-341.

21 Salas JJ, Ohlrogge JB (2002) Characterization of substrate specificity of plant FatA and FatB acyl-ACP thioesterases. Arch Biochem Biophys 403:25-34.

22 Salminen TA, Blomqvist K, Edqvist J (2016) Lipid transfer proteins: classification, nomenclature, structure, and function. Planta 244(5):971-997.

23 Todd J, Post-Beittenmiller D, Jaworski JG (1999) *KCS1* encodes a fatty acid elongase 3-ketoacyl-CoA synthase affecting wax biosynthesis in *Arabidopsis thaliana*. Plant J 17:119-130.

24 Wang Y, Wu H, Yang M (2008) Microscopy and bioinformatic analyses of lipid metabolism implicate a sporophytic signaling network supporting pollen development in *Arabidopsis*. Mol Plant 1(4):667-674.

25 Wu GZ, Xue HW (2010) *Arabidopsis* β-ketoacyl-[acyl carrier protein] synthase 1 is crucial for fatty acid synthesis and plays a role in chloroplast division and embryo development. Plant Cell. 22(11):3726-3744.
